# Nosocomial Hygiene

**Published:** 1895-11

**Authors:** Stephen Yates Howell

**Affiliations:** Gynecologist to the Erie County Hospital, Buffalo, N. Y.


### NOSOCOMIAL HYGIENE.

By STEPHEN YATES HOWELL, M. A., M. D , M. R. C. S., Eng.
Gynecologist to the Erie County Hospital, Buffalo, N. Y.

FROM the earliest times it has ever been a universally recognised fact that when a patient was isolated from all others who might be in need of the healing art, his chances of recovery were enhanced, *ceteris paribus*. And even today such a view is doubtless correct, though materially modified by the brilliant advances made since Lister first promulgated his startling doctrines in the dawn of the last quarter-century. It was on the score of convenience and with an eye to economy, chiefly, that the sick were aggregated in buildings specially arranged for their reception and care and from two or three centuries before Christ to the present day, from the primitive constructions of the Buddhist priests of Hindostan to the colossal piles of the nineteenth century, the evolution of the hospital may be traced. While the advantages of thus grouping ailing humanity were easily recognised, its evils were equally apparent and the chronicles of all these centuries teem with references to the baleful effects of crowded wards, putrid exhalations and poisoned air. In 1778, Dr. Jones, to whose efforts the New York Hospital partly owed its existence, reported the frightful condition of affairs in the large hospitals of England and France, which he had visited shortly before. One-third of all the deaths in Paris were said to have occurred in the hospitals, the old Hôtel Dieu losing annually 20 per centum of all admitted patients.

Later, the frightful mortality witnessed by Florence Nightingale caused her to doubt whether hospitals had not destroyed more lives than they had preserved and, in 1869, Sir James Y. Simpson coined the word "hospitalism" to express "the hygienic evils which the system of huge and colossal hospital edifices has hitherto been made to involve." In consequence of the terrible mortality, the destruction of all old hospital buildings and their replacement with new structures was seriously discussed in England. Instead of constructing large, imposing piles of brick and stone, it was recommended that hospitals be small fabrics of wood or iron, which could be destroyed without great pecuniary loss when they became "pyemia stricken," as Erichsen characterised it. During and subsequent to our own civil war the use of wooden barracks and of tents, for hospital purposes, was advocated by the army surgeons for the same reasons. But it is useless to elaborate this theme; most of us are familiar with it, for we have witnessed personally the old pre-Listerian and the modern post-Listerian order of things. With the discovery of the vegetable microbes and the detection of their evil mission, the curtain of ignorance which had shrouded all past ages with the darkness of despair, was lifted and a new life dawned for humanity. Physicians and surgeons ceased to theorise so vaguely concerning the nature and origin of pus, and even turned a cold shoulder upon the once "laudable" variety; the ambiguous nomenclature of a pathology founded only too largely upon doubt and ignorance gave place to the direct speech which ever characterises knowledge; the etiology of sepsis being understood, the searching cleanliness of today superseded the superficial and weak detergent efforts of the past; the microbic offenders being recognised, many extrinsic and intrinsic agencies which tend to compass their destruction, before and after their invasion of the animal organism, have been successively discovered—antiseptics, the chemotactic and phagocytic activities of embryonal cells and the immunising properties of blood-serum.

Keeping pace with this rapid advancement of scientific knowledge is the natural desire to secure to humanity all the advantages to be derived from it. Not only should every provision be made for the exclusion of pathogenic microörganisms from our homes for the sick, but the patient's powers of resistance should be enhanced in every possible way. They should be supplied with fresh air in abundance and for this purpose a constant flooding of the wards through automatic air-conduits is to be preferred to the precarious employment of mechanical devices ; suitable food and intelligent nursing should be provided ; gastro-sepsis and entero-sepsis should be combatted, and, finally, all pathological conditions and depressing influences should be removed and avoided as far as possible, in order that the sufferer's ability to cope with bacterial foes may approach that of the healthy individual as closely as possible. The environment of our patients, then, should be such as would most materially aid in their restoration to health ; in other words, it should be healthy. A healthy hospital has been defined by Mr. Simon to be one "which does not, by any fault of its own, aggravate ever so little the recovery of the persons who are properly its inmates ; and the fault of its own, through which an unhealthy hospital fails to attain the best results for its medical and surgical treatment, is of two kinds—either it is an inherent fault, as of site and construction, or else it is a fault of keeping, as dirtiness or overcrowding, or neglect of ventilation."

Now, it is by no means my intention to enter elaborately into the discussion of all the features of the perfect hospital, want of time and space both precluding such a course, but I would emphasise the fact that in the construction of the most recent examples of hospital architecture a vast amount of labor and careful thought have been expended in the endeavor to meet the manifold problems which confront the builders. I would be suggestive rather than exhaustive and to that end have chosen as my theme the subject of hygiene, the importance of which is so frequently attested by the writings of others. Carefully conducted experiments have shown that the air in mid-ocean is perfectly sterile, but as land, and particularly the habitation of man, are approached, the presence of minute forms of vegetable life in the atmosphere becomes proportionally evident. While we admit that these are usually of a non-pathogenic character in interiors where ventilation is free and where even ordinary cleanliness has been observed, and conceding that the air in such localities may be well-nigh neglected as a medium of infection, still such is not universally the case. Should defective plumbing permit the free discharge of sewer-gas into our wards and operating-rooms, trouble is sure to ensue. Sore throat, attended with fever and other evidences of systemic disturbance of variable severity supervene; our operation-wounds are invaded by pus-microbes and suppurate; in short, the familiar scenes of the old-time hospital are reënacted. The mere presence of bath-rooms, closets and sinks in our wards is a constant menace to the health of their occupants. Plumbers are proverbially incompetent or careless, and plumbing fixtures are subjected to the strains of settling walls and floors and to the wear and tear of actual use, and hence it is unreasonable to place any great reliance upon either. A defective trap, a broken joint, a cracked sewer-pipe and many other accidents of like nature, and in ofttimes unsuspected localities, afford entrance to the sewer-gas, laden with noxious germs, to the detriment of the building's occupants.

In the ideal hospital all drains should be outside the foundation-walls, while connected fixtures, such as closets, bath-rooms, sinks or basins should be in rooms which communicate with the main building or wards only through well-ventilated lobbies, corridors or galleries. The practical working of such a plan has been admirably exemplified at the Preston Retreat in Philadelphia, in which institution the freedom from puerperal sepsis, under Dr. Joseph Price's management, has been unparalleled. In the Johns Hopkins Hospital, in Baltimore, the Sloane Maternity, in New York, the St. Denis Hospital, of Paris, and the Antwerp Civil Hospital similar, though not always equal, precautions have been taken to insure the absence of any sepsis-laden effluvia from the drains.

One of the latest and most convincing articles in support of this position appeared in the January number of the *Annals of Gynecology and Pediatry*, as a contribution from the pen of Dr. A. Lapthorn Smith, of Montreal, and in view of its illustrative value I may be pardoned for the introduction of the following extended quotation:

Three years ago I took the service of a colleague who was absent on a Summer holiday, and obtained union by first intention almost invariably both in the abdominal incisions and in my operations on the cervix and perineum, and after a removal of the breast. A few months later the absent one returned, when the Autumn winds rendered it more pleasant to have the windows closed. What was the result ? Suppuration was occasionally seen in the wounds and now and then a cervix or a perineum failed to unite. In my own mind I attributed this difference in healing to my friend being a little less scrupulous in the exercise of aseptic precautions. But in this I wronged him, as it was afterward made clear. My regular term of service came round on January 1st, when the double windows were on and all the cracks were pasted up with paper or stuffed with cotton, it being difficult, even then, in very cold weather to keep the building comfortable. Taking especial pride in getting my wounds to heal without suppuration I redoubled my aseptic precautions, but they did no better than my colleague's, who had just finished his term of service. First, in a case of Alexander's operation, which at my private hospital always had healed by first intention, at the other institution the edges next day became a little red and around the stitches there was a little thickening, and a day or two later the wound was suppurating. Then I learned from my friend in charge of the obstetrical department on the top flat of the building, that in spite of every precaution he was having a series of high temperatures in every woman who was confined. Next a case of curetting and repair of the cervix and perineum under my care suddenly developed a high temperature—an almost unheard-of thing heretofore. She looked so ill and her pulse became so rapid, that a thorough examination was made, when a diphtheritic membrane was found covering the cervix and perineum. This was thoroughly cleaned with strong bichloride of mercury and frequently douched with the same, and the patient was placed on a very supporting diet. I felt sure that there was something wrong with the drainage and requested the authorities to have it examined. Several of the authorities took a different view of the cause of the outbreak, attributing it to a visitor who had a child sick with diphtheria, having visited a patient in another ward. Besides, they claimed that they had had the drainage overhauled during the previous Summer. Then several other non-operative cases developed sore throats, as did some of the resident staff. Then three cases of midwifery on the top flat developed diphtheria of the womb and vagina, one of them dying very quickly of a sort of gangrene of the uterus, another one dying a week later, both in spite of the most active treatment with peroxide of hydrogen, and the like, and a third one being cured with great difficulty.

Then all the patients were sent home and attended there by their respective surgeons and physicians, where all recovered. Then the authorities had the plumbing examined by means of the smoke test, which consists in placing a box on the roof of the building near the ventilating soil pipe, with which the smoke box is hermetically connected. Some cotton waste. impregnated with some oleoresinous material, is lighted and gives out a great quantity of pungent, yellow smoke, which is forcibly pumped down the soil pipe. until it reaches the sewer.

If there is the slightest leak of sewer-gas in any of the pipes, of course, this smoke will escape equally as well. The sewer-gas cannot be seen or even sometimes smelled, but the smoke of the smoke-box can be both seen and smelled as it pours forth from any defect. In the case under notice the pipes were found to be all staunch and faultless, but a vast stream of smoke was seen to emerge from a three-inch hole in the concrete floor of the laundry, and on further investigation it was found that this hole connected directly, without any trap whatever, with the soil pipe running into the sewer.

This was a plumber's blunder, but it cost two lives and a great many more long and tedious convalescences, both of gynecological and obstetrical cases. This defect was at once remedied and work begun again, wounds all healing, as a rule, by first intention and a high temperature in the obstetrical ward being the exception.

Two cases of severe puerperal septicemia, one in a home of wealth where great attention had been bestowed on the sanitary arrangements also occurred in Dr. Smith's practice and in each house the smoke-test disclosed plumbing defects which permitted the free ingress of sewer-gas. The greater frequency of puerperal fever during the winter months, when windows are left closed and oftentimes sealed, is a most suggestive fact in this connection and its occurrence should naturally excite our suspicion that plumbing defects were at the bottom of it. In concluding his article, Dr. Smith says :

I would, therefore, lay it down as a wise rule to follow that whenever we have suppuration of our wounds or high temperatures after confinements, notwithstanding that we have employed the most rigorous aseptic and antiseptic precautions, we should in every case suspect the plumbing, until it shall have been proved innocent, and for this no test should be accepted as sufficient except the smoke-test.

164 Franklin Street.

---

Nephropexy by Living Tendon.—M. Poullet has reported a successful case in which a tendon of the longissimus dorsi muscle was detached at its upper end from its muscular belly and passed so as to make a loop through the posterior part of the capsule of the kidney, thus supporting and holding the kidney in its proper place. This is probably the first case of the kind where a living suture was used.—*Boston Medical and Surgical Journal.*

